# Therapeutic Potential of Vericiguat in Myocardial Ischemia/ Reperfusion Injury: Crosstalk between Heat Shock Protein 90 and Complement Activation

**DOI:** 10.2174/0115665240406330250911142757

**Published:** 2025-11-19

**Authors:** Junyan Chen, Sijie Pan, Dongxiao Wang, Jianjun Meng, Min Wang, Guoqiang Zhong, Ronghui Tu

**Affiliations:** 1 Department of Cardiology, First Affiliated Hospital, Guangxi Medical University, Nanning, 530021, China;; 2 Department of Geriatric Cardiology, First Affiliated Hospital, Guangxi Medical University, Nanning, Guangxi, 530021, China;; 3 Guangxi Key Laboratory of Precision Medicine in Cardio-Cerebrovascular Diseases Control and Prevention, Nanning, 530021, Guangxi, China;; 4 Guangxi Clinical Research Center for Cardio-Cerebrovascular Diseases, First Affiliated Hospital, Guangxi Medical University, Nanning, 530021, China

**Keywords:** Vericiguat, myocardial ischemia/reperfusion injury, HSP90, complement, inflammation, apoptosis, mice

## Abstract

**Introduction:**

The present study aimed to examine the functions of heat shock protein 90 (HSP90), NF-κB, C3, and C5a in cardioprotective effects induced by vericiguat in mice.

**Methods:**

Male mice were randomly assigned to six groups: sham, ischemia/ reperfusion (I/R), vericiguat preconditioning (VPre), VPre + HSP90 inhibitor geldanamycin (GA), vericiguat postconditioning (VPost), and VPost + GA. An experimental mouse model of I/R was established in mice through surgery and treatments with vericiguat and GA. The following parameters were assessed: myocardial infarct size; cardiomyocyte apoptosis; cTnI, CK-MB, and LDH serum levels, protein expression levels of Bcl-2, Bax, HSP90, NF-κB, and complement components C3 and C5a, and mRNA expression levels of IL-1β, TNF-α, and ICAM-1.

**Results:**

Vericiguat significantly attenuated the myocardial infarct size induced by I/R injury; suppressed cardiomyocyte apoptosis; reduced serum levels of myocardial markers (CK-MB, LDH, and cTnI); decreased C5a, and C3 levels, NF-κB signaling, and expression of inflammatory cytokine （ICAM-1,TNF-α, and IL-1β); and enhanced HSP90 and Bcl-2 expression levels. However, GA reversed these effects.

**Discussion:**

The study contributes to the investigation of the crosstalk between HSP90 and complement in the protective effects of vericiguat on myocardial I/R injury. However, further in-depth research is needed to explore the underlying mechanisms of vericiguat's cardioprotective effects against myocardial I/R injury.

**Conclusion:**

HSP90 plays a crucial role in the cardioprotective effects of vericiguat, providing new insights into its mechanisms of action.

## 1 INTRODUCTION

Acute myocardial infarction (AMI) is the primary cause of mortality and disability worldwide [1]. Currently, reperfusion therapies, including percutaneous coronary intervention, coronary artery bypass grafting, and thrombolytic therapy, remain the most effective methods to treat myocardial infarction. However, reperfusion can cause additional damage to the ischemic myocardium. This phenomenon, known as ischemia/reperfusion (I/R) injury, can potentially aggravate myocardial damage [2]. Therefore, identifying methods and strategies to mitigate I/R injury remains a critical topic in treating AMI [3]. The cardioprotective effects of soluble guanylate cyclase (sGC) stimulants on I/R injuries have garnered significant attention in recent years [4].

sGC stimulants are recently discovered new drugs for treating cardiovascular diseases, and they function by activating the nitric oxide (NO)-sGC-cyclic guanosine phosphate (cGMP) pathway. The VICTORIA trial, the largest clinical study of sGC stimulants globally, revealed that vericiguat enhances outcomes in patients with heart failure and shows beneficial results in patients with coronary heart disease [5, 6]. Multiple studies have reported that vericiguat ameliorates myocardial I/R injury [4, 7]. However, the precise mechanisms through which vericiguat protects the heart during myocardial I/R injury remain incompletely understood.

Complement activation has been established as a significant contributor to myocardial I/R injury [8]. Complement components C3 and C5a initiate a cascade of pro-inflammatory cells, leading to the release of inflammatory mediators, including intercellular cell adhesion factor-1 (ICAM-1), tumor necrosis factor-α (TNF-α), and interleukin (IL)-1β [9-11]. Additionally, excessive activation of the complement system promotes the generation of C3 and C5a, which can activate the NF-κB signaling pathway to aggravate inflammation [12]. Thus, the complement/NF-κB signaling pathway could serve as a potential therapeutic target for treating I/R injury. However, it remains unclear whether vericiguat induces cardioprotective effects through the complement and NF-κB signaling pathway.

Heat shock protein 90 (HSP90) is an essential component of the molecular chaperone network and plays a crucial role in maintaining the integrity and function of multiple signaling proteins and stress response through cardioprotection [13, 14]. Previous research has established a strong relationship between HSP90 and apoptosis during pre- and postconditioning phases [15, 16]. Furthermore, recent reports have shown that HSP90-mediated pre- and post-conditioning induce cardioprotective effects by inhibiting the activation of NF-κB, C3 and C5a signaling [17]. HSP90 exhibits its significant role in the cardioprotective effects of liraglutide, pioglitazone preconditioning, and morphine postconditioning on cardiac I/R injury [18, 19]. However, it remains unclear whether the ability of HSP90 to suppress the activation of C3, C5a, and NF-κB signaling is the primary mechanism that contributes to vericiguat-induced cardioprotective effects.

In the present study, we hypothesized that the cardioprotective effects of vericiguat against myocardial I/R injury are mediated by inhibiting C3, C5a, and NF-κB signaling and suppressing inflammatory responses by up-regulating HSP90 expression. We evaluated the protein levels of NF-κB, HSP90, C3, and C5a and the mRNA levels of TNF-α, IL-1β, and ICAM-1. Additionally, we assessed serum levels of creatine kinase-MB (CK-MB), lactate dehydrogenase (LDH), cardiac troponin I (cTnI), and myocardial infarct size, cardiomyocyte apoptosis. The effects of the HSP90 inhibitor geldanamycin (GA) were also investigated.

## 2 MATERIALS AND METHODS

### 2.1 Animals

120 male C57BL/6J mice, aged 8-10 weeks (weighing 20 to 25 *g*), were purchased from the Laboratory Animal Center of Guangxi Medical University, China. The animals were housed at 25°C ± 2°C, with appropriate humidity, a standard 12-h light/dark cycle, and unrestricted access to food and water. All animal experiments complied with the National Institutes of Health Guide for the Care and Use of Laboratory Animals and were ethically approved by the Guangxi Medical University Animal Care and Use Committee (Approval NO.202310009).

### 2.2 Myocardial I/R Model

Mice were anesthetized with sodium pentobarbital (50 mg/kg, intraperitoneally) and intubated [20]. A small animal ventilator was used for positive pressure breathing. The chest was accessed through the 4th intercostal space and the left anterior descending (LAD) coronary artery was ligated using a 7-0 silk suture. Successful vascular closure was confirmed by changes in dynamic electrocardiographic ST elevation signals. Ischemia was maintained for 30 min, and the ligature was then released to allow reperfusion for 2 h. The skin of the thoracic cavity was closed using a 5-0 silk suture. Following completion of the reperfusion phase, myocardial tissue samples from the left ventricular anterior wall and blood samples were collected for subsequent analysis.

### 2.3 Experimental Groups

Mice were randomly allocated to six groups, with 20 mice per group: (1) Sham: the LAD branch alone was threaded without ligation; (2) I/R: the LAD branch was subjected to 30 min of ligation, followed by 2 h of reperfusion; (3) Vericiguat preconditioning (VPre): Mice in the I/R group were administered vericiguat (1 mg/kg) *via* caudal vein injection 12 h prior to ischemia induction [21]; (4) VPre + GA: Mice in the I/R group were intraperitoneally administered GA (1 mg/kg [22]) 30 min prior to ischemia induction. The subsequent steps followed the same protocol as that for the VPre group; (5) Vericiguat postconditioning (VPost): Vericiguat (1 mg/kg [21]) was administered by caudal vein injection 10min prior to reperfusion initiation, followed by 2 h; (6) VPost + GA: Mice in the I/R group were intraperitoneally administered GA (1 mg/kg [22]) 10 min prior to intravenous vericiguat administration.

### 2.4 Assessment of Myocardial Infarction Size

Infarct size determination was performed across all six experimental groups. Following 2 h of reperfusion, the LAD coronary artery was again occluded. Subsequently, 2% Evans blue was administered into the left heart cavity. The stained hearts were frozen, cut into 1-inch thick horizontal slices, and incubated for 15 min at 37°C in 2,3,5-triphenyltetrazolium chloride (1%; TTC, Sigma-Aldrich, USA) under light protection to determine the size of the myocardial infarct area. Digital imaging software was used to calculate both infarct size and total left ventricular area.

### 2.5 Determination of Serum Levels of LDH, CK-MB, and cTnI

Post-reperfusion blood samples were collected in 1.5 mL centrifuge tubes and maintained at room temperature for 30 min, followed by centrifugation at 3000 g for 15 min at room temperature.

The extracted supernatant was stored at -80°C. The cTnI levels were measured using a cTnI ELISA kit (Cusabio, China) in accordance with the manufacturer's instructions on an automated antibody analyzer (Hitachi 7600, Tokyo, Japan). CK-MB and LDH levels were determined using an automated biochemical analyzer (IDEXX Laboratories Inc., Maine, USA).

### 2.6 TUNEL Staining

Mice were euthanized, and their chest cavities were rapidly accessed to extract the hearts. The hearts were then cleansed with physiological saline and the left ventricular myocardial tissues were excised. Cardiac tissues were fixed with 4% paraformaldehyde, then dehydrated and embedded. A TUNEL kit (Biyun Tian, China) was used for cell staining. Apoptosis was detected by fluorescence microscopy. Five areas were randomly selected from each slide, and the ratio of apoptotic cardiomyocytes to total cardiomyocytes (apoptosis index, %) was calculated using ImageJ software.

### 2.7 Determination of mRNA Expression Levels in Heart Tissues

Total RNA was extracted from heart tissues by using Trizol reagent, and the obtained RNA was reverse transcribed with HyperScriptTM III RT SuperMix (Xinbei, China), and the polymerase chain reaction was conducted using 2×S6 Universal SYBR qPCR Mix on a 7500 real-time fluorescent quantitative PCR system. GAPDH was utilized as the reference gene in this experiment. The following corresponding primers were used [23]: IL-1β forward 5′-TGCCACCTTTTGACAGTGATGA-3′ and reverse 5′-GAGTGATACTGCCTGCCTGAAG-3'; TNF-α forward 5'-ACCACGCTCTTCTGTCTACTG-3' and reverse 5'-GAGGGTCTGGGGCCATAGAACT-3'; and ICAM-1 forward 5'-TGGGTCGAAGGTGGTTCTTCT-3' and reverse 5'-GTCCAGCCGAGGACCATACAG-3'. Relative gene expression levels were calculated using the 2 - ΔΔCt method [24].

### 2.8 Western Blot Analysis

Total protein was extracted from heart tissues by using RIPA lysate (EpiZyme, China), and protein concentration was then determined using a bicinchoninic acid kit (Solarbio, China). Following electrophoresis, the samples were transferred to polyvinylidene difluoride (PVDF) membranes, followed by membrane blocking with TBST containing 5% skimmed milk. The membranes were then incubated overnight at 4°C with the following rabbit primary antibodies: HSP90 (1:5000, Proteintech, #13171-1-AP, Wuhan, China), Bcl-2 (1:3000, Signalway Antibody, #40639, Baltimore, MD), Bax (1:2000, Abways, #CY5059, China), C3 (1:3000, Abcam, #EPR19394, Cambridge, UK), C5a (1:2000, ABclonal, #A8104, Wuhan, China), NF-κB p65 (1:5000, Abways, #CY5034, China), and GAPDH (1:2000, Servicebio, #GB15004, Wuhan, China). Horseradish peroxidase-labeled goat anti-rabbit secondary antibodies (1:10000, Proteintech, #SA00001-2, Wuhan, China) were used for 1 h of incubation at room temperature. The signals were captured using e-BLOT Touch Imager Pro, and protein expression was analyzed using ImageJ software [25].

### 2.9 Statistical Analysis

SPSS 25.0 software was used for statistical analysis of the obtained data. Measurement values were expressed as mean ± standard deviation. Mean values of multiple groups were compared by one-way analysis of variance (ANOVA). Statistical significance was considered at *P* < 0.05.

## 3 RESULTS

 A total of 120 mice were utilized in this experiment. Four mice died due to ventricular arrhythmia, cardiogenic shock, and respiratory failure: one each in the I/R, VPre + GA, VPost, and VPost + GA groups. Following exclusion and additional analysis, the obtained results were based on the remaining 116 mice.

### 3.1 Vericiguat Upregulated HSP90 Protein Expression

To determine the role of HSP90 in VPre and VPost treatments, we assessed the protein expression levels of HSP90. The results demonstrated that both VPre and VPost treatments significantly increased HSP90 protein levels compared to that in the I/R group (*P* < 0.05, Fig. **1**). Furthermore, HSP90 protein levels were decreased in the VPre + GA and VPost + GA groups. These findings indicate that VPre and VPost alleviated myocardial I/R injury by upregulating the HSP90 protein expression level.

### 3.2 Vericiguat Ameliorated I/R-induced Myocardial Infarct Size *Via* HSP90

As shown in Fig. (**2**), the sham group exhibited no myocardial infarction. The infarct sizes of the VPre and VPost groups were significantly smaller than that of the I/R group (18.38 ± 2.48% *vs*. 36.61 ± 2.01% and 24 ± 2.15% *vs*. 36.61 ± 2.01%, all *P* < 0.05). Conversely, the VPre + GA and VPost + GA groups showed a significant increase in the infarct size (18.38 ± 2.48% *vs*. 31.67 ± 1.51% and 24 ± 2.15% *vs*. 36.74 ± 1.43%, all *P* < 0.05).

### 3.3 Vericiguat Mitigated I/R Injury-induced Cardiomyocyte Apoptosis Through Protective Effects Mediated by HSP90

As depicted in Fig. (**3**), the I/R group exhibited numerous apoptotic cells, with an apoptosis rate of 59.97 ± 11.11%. The VPre and VPost groups showed significantly reduced apoptosis rates of 15.75 ± 4.32% and 16.53 ± 3.2%, respectively, compared to the I/R group (all *P*< 0.05). However, GA inhibited the anti-apoptotic effects of VPre and VPost (apoptosis rates for the VPre + GA and VPost + GA groups: 35.45 ± 4.17% and 38.22 ± 5.83%, respectively, all *P* < 0.05).

To investigate the mechanisms through which VPre and VPost inhibit apoptosis, we determined the protein expression levels of Bcl-2 and Bax. Compared to the sham group, the I/R group exhibited a notable increase in the Bax protein level, and a marked reduction in the Bcl-2 protein level (*P* < 0.05, Fig. **4**). The VPre and VPost groups demonstrated significantly elevated, Bcl-2 protein levels and downregulated Bax protein levels (*P* < 0.05). These expression patterns were reversed in the VPre + GA and VPost + GA groups.

### 3.4 Vericiguat Ameliorated I/R-induced Myocardial Injury *Via* HSP90

 Compared to the sham group, serum levels of CK-MB, LDH, and cTnI were markedly elevated in the I/R group (Table **1**). The VPre and VPost groups exhibited a significant reduction in serum levels of CK-MB (744.8 ± 123.6 *vs*. 1623.2 ± 212.0 and 827.4 ± 161.2 *vs*. 1623.2 ± 212.0, all *P* < 0.05), cTnI (22.4 ± 5.9 *vs*. 70.5 ± 4.7 and 33.6 ± 7.9 *vs*. 70.5 ± 4.7, all *P* < 0.05), and LDH (1687.4 ± 75.1 *vs*. 2982.8 ± 372.2 and 1560.4 ± 309.7 *vs*. 2982.8 ± 372.2, all *P* < 0.05) compared to those in the I/R group. These results indicate that VPre and VPost treatments effectively reduced myocardial injury induced by I/R.

### 3.5 Vericiguat Alleviated I/R-induced Inflammatory Responses *Via* HSP90

 Considering the pivotal role of inflammation in myocardial I/R injury, RT-qPCR was conducted to measure the mRNA expression levels of IL-1β, TNF-α, and ICAM-1. Compared to the I/R group, the sham group displayed a significant decrease in the mRNA expression levels of IL-1β, TNF-α, and ICAM-1 (all *P* < 0.05, Fig. **5**). However, the I/R, VPre + GA, and VPost + GA groups demonstrated significantly higher expression levels of IL-1β, TNF-α, and ICAM-1 compared to the VPre and VPost groups (all *P* < 0.05).

### 3.6 Vericiguat Mitigated I/R Injury-induced Activation of C3, C5a, and NF-κB Through a HSP90-mediated Mechanism

We evaluated the relationship between HSP90 and the C3, C5a, and NF-κB pathways under VPre and VPost treatments by using the HSP90 inhibitor GA. VPre and VPost treatments decreased the levels of C3, C5a, and NF-κB compared to those in the I/R group (all *P* < 0.05). GA administration reversed the inhibitory effects of VPre and VPost on C3, C5a, and NF-κB (all *P* < 0.05, Fig. **6**). These findings suggest that HSP90 mediates the cardioprotective effects of VPre and VPost by inhibiting C3 and C5a activation and suppressing NF-κB signaling. The results indicate that VPre and VPost exert their cardioprotective effects by inhibiting complement system activation and NF-κB signaling. Furthermore, these effects of VPre and VPost were closely associated with HSP90 activity.

## 4 DISCUSSION

The key finding of the present study is that vericiguat substantially attenuated myocardial I/R injury through HSP90-mediated mechanisms. We demonstrated that vericiguat markedly reduced the infarct area, decreased the extent of cardiomyocyte apoptosis, and lowered serum levels of LDH, CK-MB, and cTnI. Additionally, vericiguat reduced the expression levels of C3, C5a, NF-κB, and Bax and decreased the release of inflammatory factors such as TNF-α, IL-1β, and ICAM-1. Concurrent with these beneficial effects, the expression levels of HSP90 and Bcl-2 were upregulated. However, the introduction of the HSP90 inhibitor GA negated the cardioprotective effects of vericiguat. Thus, these findings highlight the crucial role of HSP90 in vericiguat-induced cardioprotection, potentially through the inhibition of C3, C5a, and NF-κB activation.

Vericiguat, a novel sGC stimulator, is currently utilized for treating heart failure. Based on the largest sGC stimulator clinical trial published to date, vericiguat demonstrated exceptional efficacy in treating heart failure patients with coronary heart disease, which suggests its potential for treating I/R injury [6]. Previous animal studies have demonstrated that vericiguat ameliorates myocardial I/R injury by enhancing microcirculation, promoting angiogenesis, improving autophagy, and exhibiting anti-apoptotic effects [4, 7]. Thus far, few studies have investigated whether vericiguat counteracts myocardial I/R injury through HSP90 during pre- and post-conditioning treatments. Therefore, by utilizing an *in* *vivo* I/R mouse model, this study revealed that vericiguat pre- and post-conditioning significantly elevated HSP90 expression to protect the myocardium from I/R injury. However, the HSP90 inhibitor GA reversed vericiguat-induced cardioprotective effects.

Cardiomyocyte apoptosis plays a crucial role in myocardial I/R injury [26-28]. The survival of cardiomyocytes in the ischemic area is dependent on the ratio between the anti-apoptotic protein (Bcl-2) and the pro-apoptotic protein (Bax) [29]. An increase in the Bcl-2 expression level modulates cardiomyocyte apoptosis by inhibiting the synthesis of Bax [30, 31]. We previously reported that an increased Bcl-2 expression level in ischemic postconditioning depended on HSP90; this process was accompanied by reduced Bax expression [22]. Furthermore, previous studies demonstrated that treatment with the HSP90 inhibitor GA blocked the interaction of HSP90 with Bcl-2, thereby promoting mast cell apoptosis [32]. In the present study, we found that combining vericiguat with GA neutralized VPre- and VPost-induced elevation in Bcl-2 expression. This finding implies that the anti-apoptotic effect of vericiguat is closely associated with HSP90 expression.

Complement system activation plays a vital role in the pathogenesis of myocardial I/R injury [9]. Complement components C3 and C5a, generated through the activation of the classical, lectin, and alternative complement system pathways, accumulate in the hypoxic myocardium; these accumulated complement components exacerbate myocardial necrosis and facilitate leukocyte chemotaxis to trigger the release of inflammatory cytokines (ICAM-1, TNF-α, and IL-1β), eventually resulting in myocardial damage [33, 34]. Furthermore, C5a enhances the inflammatory response by activating NF-κB signaling [12]. Besides vericiguat, there are other sGC stimulators such as cinaciguat. Additionally, a previous study reported that praliciguat can decrease the levels of inflammation-related markers in I/R injury [35, 36]. To date, there are few reports on whether vericiguat can protect the myocardium from I/R injury by inhibiting TNF-α, ICAM-1, IL-1β, C3, and C5a. Our findings revealed a reduction in the levels of these inflammatory markers in both VPre and VPost groups compared to those in the I/R group. These results highlight the potential mechanisms through which vericiguat induces inflammatory suppression and cardioprotective effects.

The NF-κB signaling pathway serves an essential function in myocardial I/R injury [37]. As a crucial inflammatory response regulator, it modulates the complement component C3 to enhance complement system-mediated immune-inflammatory response [38, 39]. Additionally, NF-κB promotes the expression of proinflammatory cytokines, responds to complement activation, and exacerbates myocardial injury [12, 40, 41]. However, the interplay between complement components and NF-κB signaling in the cardioprotective effects of vericiguat remains unclear. Our results showed that vericiguat concurrently inhibits NF-κB and complement components C3 and C5a, suggesting a potential link between NF-κB signaling inhibition and complement system regulation.

HSP90 is an endogenous stress protein that primarily functions as a molecular chaperone [42]. As a companion of sGC, HSP90 facilitates the maturity of the precursor into sGC, maintains sGC stability, and enhances the production of cGMP [43-46]. HSP90 significantly contributes to the cardioprotective effects of vericiguat. In response to cellular stress and ischemic damage, the overexpression of HSP90 promotes cell survival and protects the myocardium from I/R injury during ischemic postconditioning and pharmacological conditioning [22, 47, 48]. Therefore, we conducted a study to investigate the relationship between vericiguat and HSP90 in ameliorating myocardial I/R injury. HSP90 protects cells against complement-mediated cell death [49, 50]. Previous research has reported the role of HSP90 in inhibiting C3 and C5a activation, and NF-κB signaling during pre- and post-conditioning [18, 51].

GA, an HSP90 inhibitor, binds to HSP90 and prevents its activity, leading to the degradation of its substrate proteins [52, 53]. Previous studies have shown that GA derivatives, such as 17DMAG, can inhibit HSP90 gene expression [54, 55]. In the present study, GA was utilized to examine the role of HSP90 in inhibiting C3 and C5a activation and NF-κB signaling during vericiguat-induced cardioprotection. The results revealed elevated HSP90 levels and reduced C3, C5a, and NF-κB levels in both VPre and VPost groups. However, GA treatment reversed these beneficial effects of vericiguat. To the best of our knowledge, this study presents the first evidence of the involvement of HSP90 in the inhibition of complement activation by vericiguat, providing a novel perspective for myocardial I/R injury treatment.

These findings substantiate the role of HSP90 in inhibiting C3, C5a, NF-κB, and Bax during VPre and VPost treatments. Furthermore, our observations revealed a novel mechanism of cardioprotective effects of vericiguat, including but not limited to the reduction of myocardial infarct size and cardiomyocyte apoptosis; induction of anti-inflammatory effects; and inhibition of C3, C5a, and NF-κB. To our knowledge, this study demonstrated for the first time the interaction between HSP90, Bcl-2, C3, C5a, NF-κB, and Bax in VPre and VPost treatments, indicating that vericiguat-induced cardioprotective effects are closely linked to HSP90 and the complement system.

## 5 STUDY LIMITATIONS

The study has some limitations. First, while our data provide preliminary insights into the role of HSP90 in vericiguat-induced cardioprotective mechanisms, additional research is needed to further elucidate the detailed molecular interactions and the involved signaling pathways, as well as to identify broader therapeutic targets of vericiguat in myocardial I/R injury. Second, other heat shock proteins, including HSP70 and HSP60, also participate in vericiguat-induced cardioprotection, and further studies regarding their roles are required. Third, this experiment utilized only the HSP90 inhibitor GA. Future investigations would benefit from exploring the impact of over- or under- expressing HSP90 through viral vectors to obtain a more comprehensive understanding of the role of HSP90 in VPre and VPost treatments.

## CONCLUSION

In conclusion, HSP90 plays an essential role in vericiguat-induced cardioprotection against I/R injury, partially by inhibiting complement system activation, NF-κB signaling, and Bax expression, and reducing myocardial inflammation, eventually minimizing I/R-induced myocardial infarct size, cardiomyocyte apoptosis, and myocardial injury. These findings provide novel insights into vericiguat-mediated cardioprotection and suggest potential therapeutic targets for treating I/R injury.

## AUTHORS’ CONTRIBUTIONS

The authors confirm their contribution to the paper as follows: Writing the Paper: JC; Data Collection: SP; Writing-Original Draft Preparation: DW; Data Analysis or Interpretation: JM, MW; Writing-Reviewing and Editing: GZ, RT. All authors reviewed the results and approved the final version of the manuscript

## Figures and Tables

**Fig. (1):**
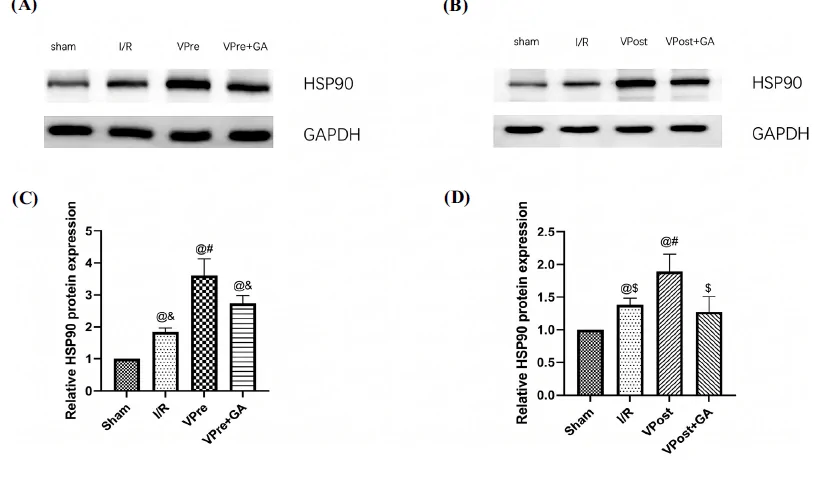
Impacts of VPre and VPost on the protein expression of HSP90. (**A**) and (**B**) display representative Western blot results illustrating the expression pattern of HSP90. (**C**) and (**D**) present the relative expression levels of HSP90 protein. The data are shown as the mean ± standard deviation. A symbol '@' indicates *P* < 0.05 when compared with the sham group; '#' represents *P* < 0.05 versus the I/R group; '&' denotes *P* < 0.05 in contrast to the VPre group; '$' shows *P* < 0.05 compared with the VPost group. There are n = 5 animals in each group.

**Fig. (2):**
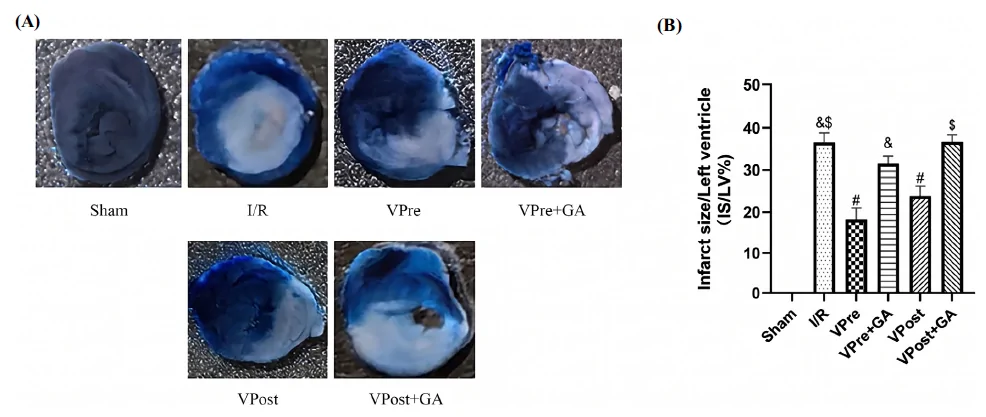
(**A**) depicts the impacts of VPre and VPost, both with and without GA, on the infarct size following myocardial I/R injury. (**B**) shows the quantitative assessment of myocardial infarct size. The outcomes are displayed as the means ± standard deviation. A '#' indicates *P* < 0.05 when compared with the I/R group; an '&' represents *P* < 0.05 versus the VPre group; a '$' denotes *P* < 0.05 compared with the VPost group. There are n = 5 animals in each group.

**Fig. (3):**
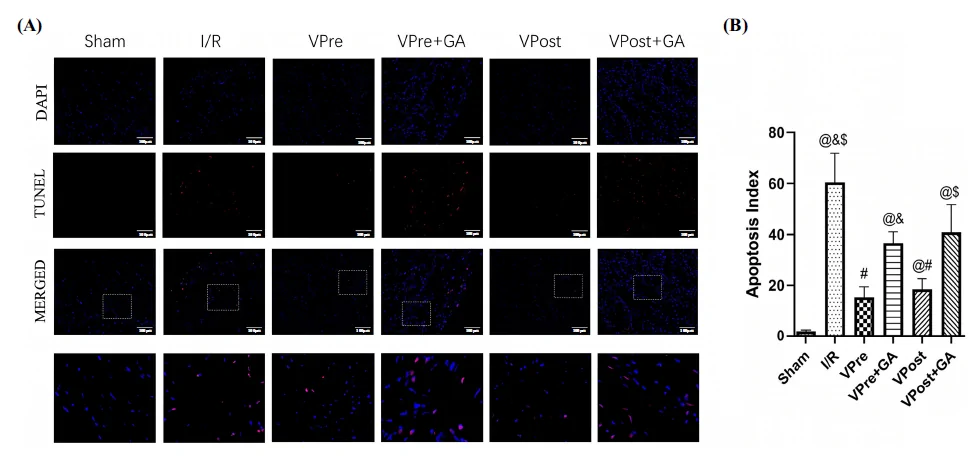
(**A**) displays the impacts of VPre and VPost, either with or without GA, on apoptosis following myocardial I/R injury. (**B**) presents the quantitative analysis of the cell apoptotic rate. The results are shown in a bar graph as the means ± standard deviation. Blue fluorescence represents DAPI staining, while red fluorescence indicates TUNEL staining. The TUNEL-stained images are at a magnification of ×200, and all scale bars are 100 μm. '@' is used to show *P* < 0.05 in comparison to the sham group; '#' is for *P* < 0.05 when contrasted with the I/R group; '&' implies *P* < 0.05 versus the VPre group; '$' denotes *P* < 0.05 compared with the VPost group. Each group consists of n = 5 samples.

**Fig. (4):**
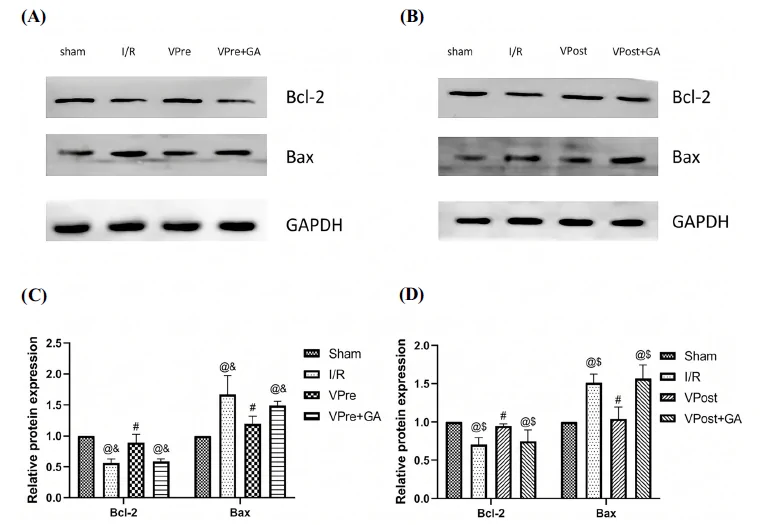
Impacts of VPre and VPost, either with or without GA, on the protein expression of Bcl-2 and Bax following myocardial I/R injury. (**A**) and (**B**) display representative Western blot results illustrating the expression patterns of Bcl-2 and Bax. (**C**) and (**D**) present the relative expression levels of Bcl-2 and Bax proteins. The data are shown as the means ± standard deviation. '@' is used to indicate *P* < 0.05 in comparison to the sham group; '#' is for *P* < 0.05 when contrasted with the I/R group; '&' implies *P* < 0.05 versus the VPre group; '$' denotes *P* < 0.05 compared with the VPost group. Each group consists of n = 5 samples.

**Fig. (5):**
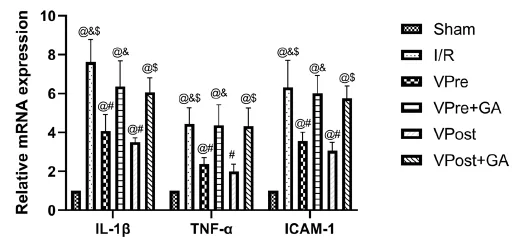
Impacts of VPre and VPost, either with or without GA, on the expression of IL-1β, TNF-α, and ICAM-1 following myocardial I/R injury. The mRNA expression levels of these three genes were determined through RT-qPCR across different groups. The results are presented as the means ± standard deviation. '@' is used to show *P* < 0.05 in comparison to the sham group; '#' is for *P* < 0.05 when contrasted with the I/R group; '&' implies *P* < 0.05 versus the VPre group; '$' denotes *P* < 0.05 compared with the VPost group. Each group consists of n = 5 samples.

**Fig. (6):**
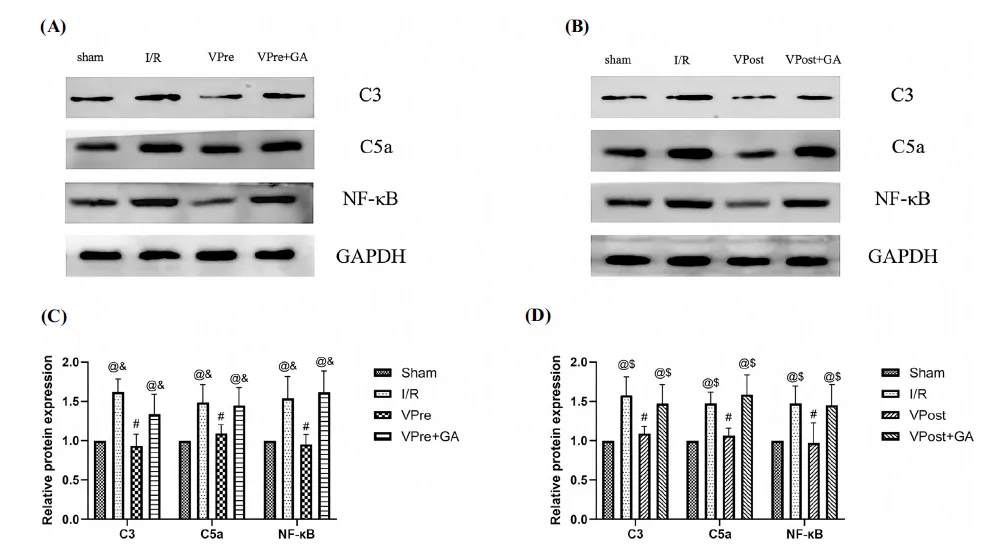
Impacts of VPre and VPost, either with or without GA, on the protein expression of C3, C5a, and NF-κB. (**A**) and (**B**) display representative Western blot results illustrating the expression patterns of C3, C5a, and NF-κB. (**C**) and (**D**) present the relative expression levels of these three proteins. The values are expressed as the means ± standard deviation. '@' is used to indicate *P* < 0.05 in comparison to the sham group; '#' is for *P* < 0.05 when contrasted with the I/R group; '&' implies *P* < 0.05 versus the VPre group; '$' denotes *P* < 0.05 compared with the VPost group. Each group consists of n = 5 samples.

**Table 1:** Serum levels of cTnI, CK-MB, and LDH in mice.

**Groups**	**CK-MB (U/L)**	**LDH (U/L)**	**cTnI (ng/mL)**
Sham	189.2 ± 43.4	528.4 ± 156.7	0.04 ± 0.02
I/R	1623.2 ± 212.0^@&$^	2982.8 ± 372.2^@&$^	70.5 ± 4.7^@&$^
VPre	744.8 ± 123.6^#^	1687.4 ± 75.1^@#^	22.6 ± 5.8^@#^
VPre + GA	1969.2 ± 491.6^@&^	2921.8 ± 351.5^@&^	79.6 ± 6.3^@&^
VPost	827.4 ± 161.2^@#^	1560.4 ± 309.7^@#^	33.6 ± 7.9^@#^
VPost + GA	1629.4 ± 289.7^@$^	2823.6 ± 208.0^@$^	70.5 ± 8.4^@$^

**Note:** The data are shown as the means ± standard deviation. '@' is used to indicate *P* < 0.05 in comparison to the sham group; '#' is for *P* < 0.05 when contrasted with the I/R group; '&' implies *P* < 0.05 versus the VPre group; '$' denotes *P* < 0.05 compared with the VPost group. Each group consists of n = 5 samples.

## Data Availability

All data generated or analyzed during this study are included in this published article.

## LIST OF ABBREVIATIONS

AMI: Acute myocardial infarction
ANOVA: Analysis of variance
cGMP: Cyclic guanosine phosphate
CK-MB: Creatine kinase-MB
cTnI: Cardiac troponin I
GA: Geldanamycin
HSP90: Heat shock protein 90
I/R: Ischemia/reperfusion
ICAM-1: Intercellular cell adhesion factor-1
IL: Interleukin
LAD: Left anterior descending
LDH: Lactate dehydrogenase
NO: Nitric oxide
PVDF: Polyvinylidene difluoride
sGC: Soluble guanylate cyclase
TNF-α: Tumor necrosis factor-α
VPost: Vericiguat postconditioning
VPre: Vericiguat preconditioning
